# Photochemically Controlled Drug Dosing from a Polymeric Scaffold

**DOI:** 10.1007/s11095-017-2164-9

**Published:** 2017-05-15

**Authors:** Louise Donnelly, John G. Hardy, Sean P. Gorman, David S. Jones, Nicola J. Irwin, Colin P. McCoy

**Affiliations:** School of Pharmacy, Queen’s University Belfast, Belfast, Northern Ireland BT9 7BL UK

**Keywords:** Controlled drug release, Dosing, Hydrogel, Light, pMEA

## Abstract

**Purpose:**

To develop the first photoactive biomaterial coating capable of controlled drug dosing via inclusion of synthesised drug-3,5-dimethoxybenzoin (DMB) conjugates in a poly(2-methyoxyethyl acrylate) (pMEA) scaffold.

**Methods:**

Flurbiprofen- and naproxen-DMB conjugates were prepared via esterification and characterised via NMR spectroscopy and mass spectrometry following chromatographic purification. Conjugate photolysis was investigated in acetonitrile solution and within the pMEA matrix following exposure to low-power 365 nm irradiation. Photo-liberation of drug from pMEA into phosphate buffered saline was monitored using UV-vis spectroscopy.

**Results:**

The synthetic procedures yielded the desired drug conjugates with full supporting characterisation. Drug regeneration through photolysis of the synthesised conjugates was successful in both acetonitrile solution and within the pMEA scaffold upon UV irradiation. Conjugates were retained within the pMEA scaffold with exclusive drug liberation following irradiation and increased drug dose with increasing exposure. Multi-dosing capacity was demonstrated though the ability of successive irradiation periods to generate further bursts of drug.

**Conclusion:**

This study demonstrates the first application of photochemically controlled drug release from a biomaterial coating and the feasibility of using pMEA as a scaffold for housing the photoactive drug-DMB conjugates.

## Introduction

Controlled drug delivery devices offer the potential to maximize the therapeutic activity of drugs while minimizing their side effects (1). To meet this objective, the ability to target a drug to its intended site of action and maintain its release rate within the therapeutic range over an extended time period is fundamental (2). Since Wichterle and Lim developed the first hydrogel comprised of 2-hydroxyethyl methacrylate (HEMA) and ethylene dimethacrylate in the 1950’s (3), which later found widespread use as a contact lens material (4), the use of hydrogels in the pharmaceutical (5) and biomedical fields (6) has increased. Tanaka’s work on the temperature induced phase transition of polyacrylamides (7) subsequently promoted the development of stimuli-responsive hydrogels with applications as sensors, switches, artificial muscles and drug delivery devices amongst many others are currently being investigated (8). Although the mechanical fragility of hydrogels, particularly when in the water-swollen state, can limit their use as standalone medical devices, they are still useful for biomedical coatings. In this context, they have been investigated to protect implanted glucose sensors from biofouling (9) and to provide an encrustation resistant surface on urinary catheters (10). In addition to improving lubricity and biocompatibility of the implantable device, the addition of a hydrogel layer allows incorporation of active agents and subsequent drug releasing capabilities from the material.

The ability to couple the application of a stimulus to the liberation or release of a from delivery devices upon application of stimuli is an attractive concept, allowing high levels of pharmaceutical control which may not be easily achieved through conventional delivery modes. In the case of drug delivery systems utilising light as an external trigger, therapeutic control can be both of a temporal and spatial nature and with the ability to influence intensity, wavelength and duration of light exposure, the amount of drug released can be manipulated.

**Fig. 1:**
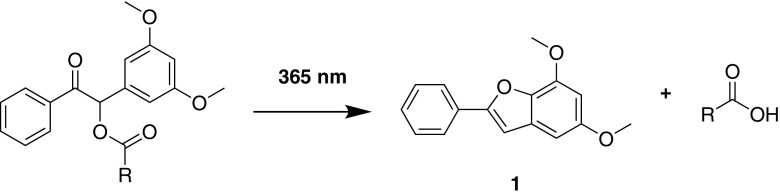
Photochemical deprotection of DMB esters.

McCoy and coworkers have previously reported the use of a common protecting group in organic synthesis, 3,5-dimethoxybenzoin (DMB), to synthesize photosensitive drug-conjugates through esterification of model drug compounds (11,12). Photoactivity was demonstrated in acetonitrile solution following exposure to low power, minimally damaging 365 nm UV-A radiation. The UV-vis absorption spectra of irradiated solutions illustrated the depletion of the conjugate and simultaneous liberation of drug and formation of a photochemical by-product 5,7-dimethoxy-2-phenylbenzofuran (1) as depicted in Fig. 1.

**Fig. 2:**
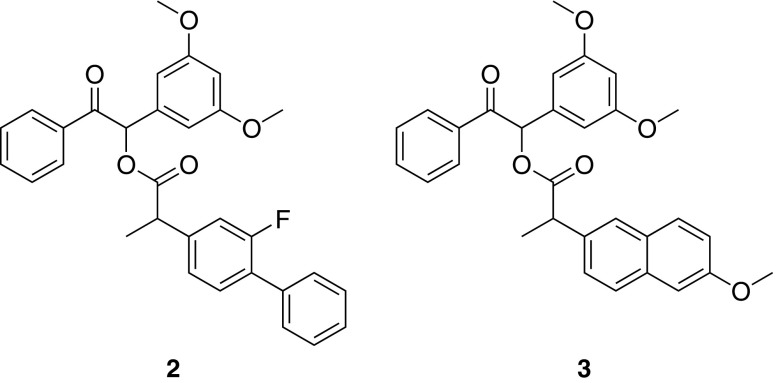
Structures of flurbiprofen-DMB ester (**2**) and naproxen- DMB ester (**3**).

In this study, we investigate the synthesis of two further drug-conjugates of DMB prepared from two non-steroidal anti-inflammatory drugs (NSAIDs), namely flurbiprofen and naproxen. Chronic use of these medications, which are used during eye surgery and for the treatment of rheumatoid arthritis, ankylosing spondylitis, and degenerative joint disease for the reduction of pain, fever and inflammation (13), has shown efficacy for treatment of gastric or duodenal ulcers in patients. Moreover, masking the free carboxylic group has been shown to improve gastrointestinal tolerability (14). It is this carboxylic acid group which lends itself to facile esterification with DMB for formation of the light-sensitive conjugates which are depicted in Fig. 2.

Here, we incorporate our synthesized drug conjugates into poly(2-methoxyethyl acrylate) (pMEA) in order to demonstrate the first application of light-triggered release from a biomaterial coating. Two aspects of drug release were considered, firstly, the ability to regulate drug release by changing the longevity of light exposure and secondly, the ability to release multi-doses of drug by multiple exposures to UV irradiation.

## Materials and Methods

### Materials

Flurbiprofen, naproxen, anhydrous dichloromethane (DCM), dimethylaminopyridine (DMAP), dicyclohexylcarbodiimide (DCC), dimethylformamide (DMF) ethylene glycol dimethacrylate, 2-methoxyethyl acrylate, phosphate buffer tablets (pH 7.3 ± 0.2 @ 25°C), silica, silica plates and triethylamine were all purchased from Sigma-Aldrich (Gillingham, Dorset, UK). All other chemicals were of AnalaR or equivalent grade and purchased from BDH laboratories (Poole, Dorset, UK).

### Synthesis and Characterisation of Photoconjugates

The synthesis of DMB was carried out as previously reported in the literature (11,12) and all procedures were carried out under protection from light. Nuclear magnetic resonance spectra were recorded on a Bruker 400 Ultrashield Plus instrument with the chemical shifts reported as parts per million (ppm) as referenced to tetramethylsilane (TMS) as an internal standard. Mass spectra were recorded on a Thermofisher LTQ Orbitrap XL in electrospray mode.

### 1-(3,5-Dimethoxyphenyl)-2-Oxo-2-Phenyl-Ethyl] 2-(3-Fluoro-4-Phenyl-Phenyl)Propanoate (2)

Compound **2** was synthesized via DCC mediated esterification. Flurbiprofen (0.82 g, 3.35 mmol), DMB (1.01 g, 3.7 mmol), and DMAP (45 mg, 0.37 mmol) were dissolved in anhydrous DCM (50 ml) with stirring prior to the addition of DCC. The mixture was stirred at room temperature overnight during which time dicyclohexylurea precipitated from solution. The filtrate was washed with 0.2 M HCl, saturated sodium bicarbonate solution and brine prior to drying over magnesium sulfate. The filtrate was concentrated using rotary evaporation and the product purified by flash chromatography on a silica gel column using DCM as the eluent. The product was isolated as a white solid (1.13 g, 67.7%). δ(CDCl_3_): 7.94 & 7.88 (d, 2H, *J* = 8.16 & 8.08, 2 x O = C-C-CH); 7.597–7.316 (m, 9H, aromatic H); 7.234–7.101 (m, 2H, aromatic H); 6.74 & 6.72 (s, 1H, O = C-CH-O-); 6.56 & 6.53 (s, 2H, 2 x -CH-C-O-CH_3_); 4.016–3.872 (m, 1H, −CH-CH_3_); 1.61 (dd, 3H, −CH_3_). m/z (%): 499.19 (M + 1, 100), 255.10 (20).

### 1-(3,5-Dimethoxyphenyl)-2-Oxo-2-Phenyl-Ethyl] 2-(6-Methoxy-2-Naphthyl)Propanoate (3)

Compound **3** was synthesized via esterification following acylation of naproxen. Naproxen (0.69 g, 3 mmol) in anhydrous DCM (ca. 15 ml) was added dropwise to thionyl chloride (0.26 ml, 3.6 mmol) and DMF (2 drops). The mixture was refluxed (3 h) and evaporated down to yield a yellow solid. Anhydrous DCM was used to resuspend the solid before adding dropwise to a solution of DMB (0.826 g, 3 mmol), and triethylamine (0.7 ml, 5 mmol) in anhydrous DCM (ca. 20 ml). The mixture was stirred at ambient temperature overnight. The brown solution was diluted with further DCM and washed with 0.2 M HCl, saturated sodium bicarbonate solution and brine prior to drying over magnesium sulfate. The filtrate was concentrated by rotary evaporation and the product purified using flash chromatography on a silica gel column using DCM as the eluent. The product was isolated as a white solid (0.56 g, 38.5%). δ(CDCl_3_): 7.92 & 7.84 (d, 2H, *J* = 8.4. & 8.8 Hz, 2 x O = C-C-CH-); 7.73–7.65 (m, 3H, aromatic H); 7.54–7.4 (m, 3H, aromatic H); 7.39 & 7.28 (t, 2H, *J* = 7.4 & 8.0 Hz, 2 x O = C-C-CH-CH-); 7.16–7.08 (m, 2H, aromatic H); 6.71 & 6.69 (s, 1H, O = C-CH-O-); 6.51 & 6.47 (d, 2H, *J* = 2.24 & 2.24 Hz, 2 x -CH-C-O-CH_3_); 6.37 & 6.34 (t, 1H, *J* = 2.28 & 2.28 Hz, −O-C-CH-C-O-); 4.1–3.99 (m, 1H, −CH-CH_3_); 3.92 & 3.91 (s, 3H, −O-CH_3_); 3.69 & 3.67 (s, 6H, 2 x -O-CH_3_); 1.65 & 1.63 (d, 3H, *J* = 7.16 & 7.2 Hz, CH_3_). m/z (%): 502.2218 (100), 485.1956 (M + 1, 61), 255.1019 (9), 232.2022 (15).

### Photolysis of Photoconjugates in Solution

Solutions of 2 and 3 (2 x 10^−5^ M) were prepared in acetonitrile and irradiated using a 15 W Hg discharge UV lamp source at 365 nm in a 1 cm path length quartz cuvette maintained at a distance of 10 mm from the light source. UV-visible absorption spectra were collected between 200–400 nm using a Perkin Elmer Lambda 650 UV-visible spectrophotometer interfaced with UV WinLab software at recorded time intervals during the irradiation period.

### Preparation of Drug-DMB Loaded pMEA

Conjugate loaded polymers were prepared by mixing MEA, the appropriate drug-DMB conjugate (5% *w*/w), EGDMA (1% *w*/w) and AIBN (0.5% *w*/w) with a magnetic stirrer until dissolution was complete. Solutions were injected between molds prepared from two glass plates lined with silicone-coated release liner, separated by silicone tubing (3 mm internal diameter, 18 mm wall thickness) and clamped together with bulldog clips. The molds were heated at 60°C for 18 h. Polymer sheets were then removed from the mould and soaked in phosphate buffered saline (PBS) for 7 days to remove any unreacted monomer. During this period, no detectable conjugate release was observed by UV-visible spectroscopy.

### Characterization of Drug-DMB Loaded pMEA

Polymer samples were prepared in the PBS swollen state with the use of a cork borer (10 mm diameter). All subsequent irradiation experiments were carried out on samples housed in PBS using a 15 W Hg discharge UV lamp kept at a fixed distance of 30 mm from the polymer samples under study.

For dose regulation studies, conjugate loaded pMEA samples were irradiated for a total of 10 or 30 min with samples being flipped to expose the lower surface at the halfway point while immersed in PBS (10 ml). Solutions were retained and samples suspended on needles which were transferred individually to universal containers housing fresh PBS and placed into an oscillating water bath at 37°C. At recorded time intervals, samples were removed and transferred to fresh buffer solutions in order to maintain sink conditions while retaining the previous solution for analysis. Retained solutions were analysed by UV-vis spectroscopy (using wavelengths of maximum absorption of flurbiprofen and naproxen of 246 nm and 261 nm respectively) and concentrations of liberated drug calculated against a standard calibration curve.

$$ Flurbiprofen\  standard\  curve: y=0.101 x+0.0116\  R2=0.9999\ \left( up\  to\ 25\mu g/ ml\right) $$

$$ Naproxen\  standard\  curve: y=0.0224 x-0.0019\  R2=0.9997\ \left( up\  to\ 50\ \mu g/ ml\right) $$

For multi-dose capacity studies, samples were treated as before with a total of 10 min of irradiation exposure followed by storage at 37°C in an oscillating water bath and assessment of drug release. When drug was no longer detectable in the release media, samples were removed from needles and re-exposed to a further period of irradiation (10 min) and subsequent drug release assessment. The irradiation and release cycle was completed a total of 5 times.

To characterise the stability of the conjugate in the pMEA matrix, samples were similarly immersed in PBS and UV-visible spectra obtained of the PBS solution every 24 h over a period of 7 days. The absorbance values at wavelengths where the relevant chromophores for flurbiprofen and naproxen absorb (246 nm and 261 nm respectively) showed no detectable absorbance, indicating no release of the conjugate from the matrix in the absence of irradiation.

## Results

### Synthesis and Characterisation of Photoconjugates

Flurbiprofen and naproxen esters of DMB were synthesised via DCC and acyl chloride mediated esterifications of the parent drug respectively according to the aforementioned synthetic procedures. **2** and **3** were isolated as white solids in 67.7% and 38.5% yield, respectively. The structures of the molecules was assigned using NMR spectroscopy and mass spectrometry.

### Photolysis of Photoconjugates in Solution

**Fig. 3:**
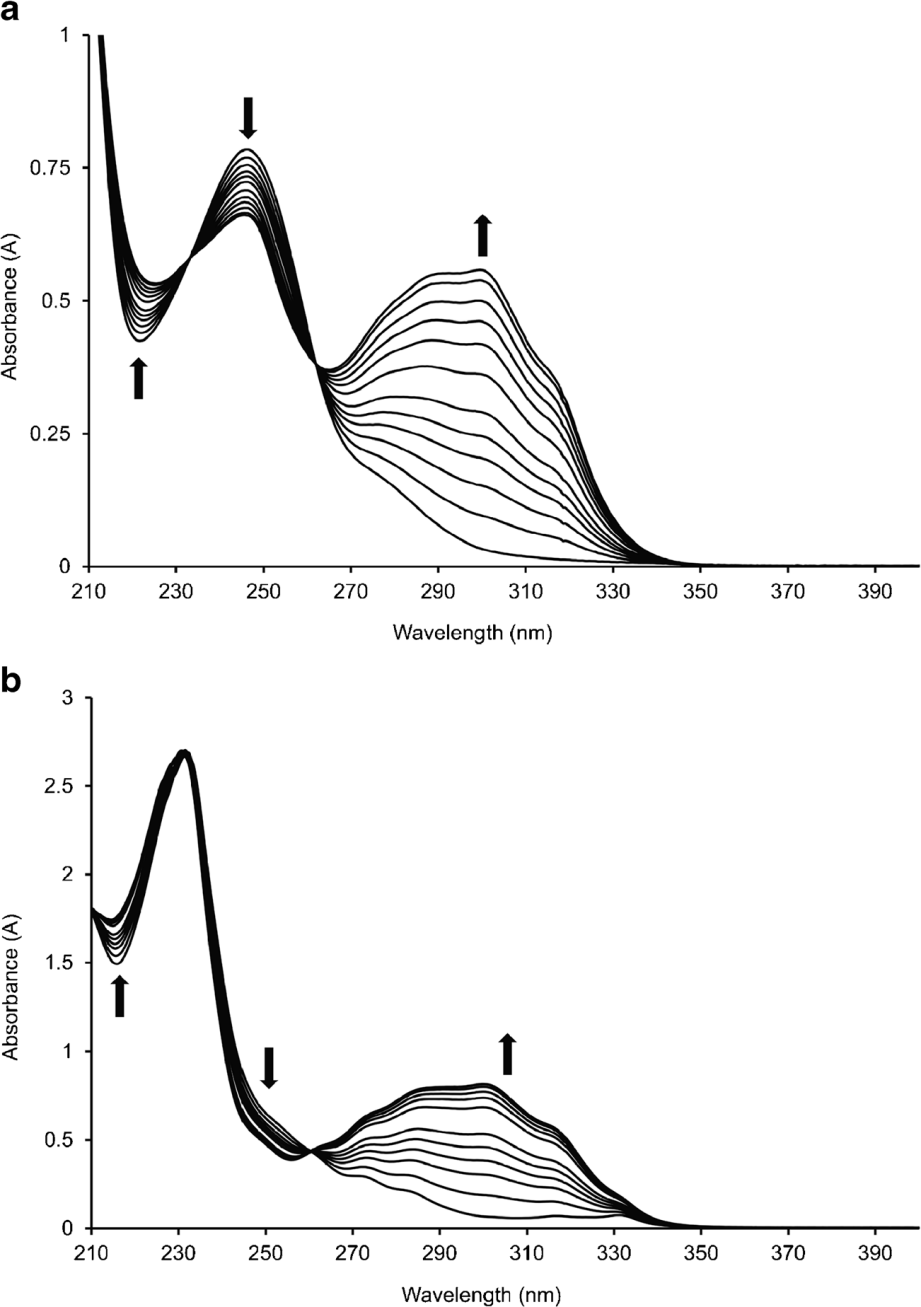
Overlaid representative UV-vis spectra of (**a**) **2** and (**b**) **3** in acetonitrile (2 x 10^−5^ M) after various periods of 365 nm irradiation. Arrows indicate absorbance trends with time and irradiation times used were 0, 30, 60, 90,120,150, 240, 300, 360,450, 570, and 690 s, respectively.

Conjugate photolysis was assessed in acetonitrile and Fig. 3 illustrates representative overlaid spectra corresponding to a) **2** and b) **3** photolysis respectively. The rates of photolysis of **2** and **3** in acetonitrile were calculated from plots of ln[A]_t_/[A]_0_, where [A]_0_ and [A]_t_ are the concentrations of drug-conjugate initially and at time t, as a function of time. Determination of the gradient, corresponding to –k, allowed calculation of the reaction rates as 4.6 x 10^−3^ and 8 x 10^−3^ s^−1^ respectively.

### Characterisation of Drug-DMB Loaded pMEA

**Fig. 4:**
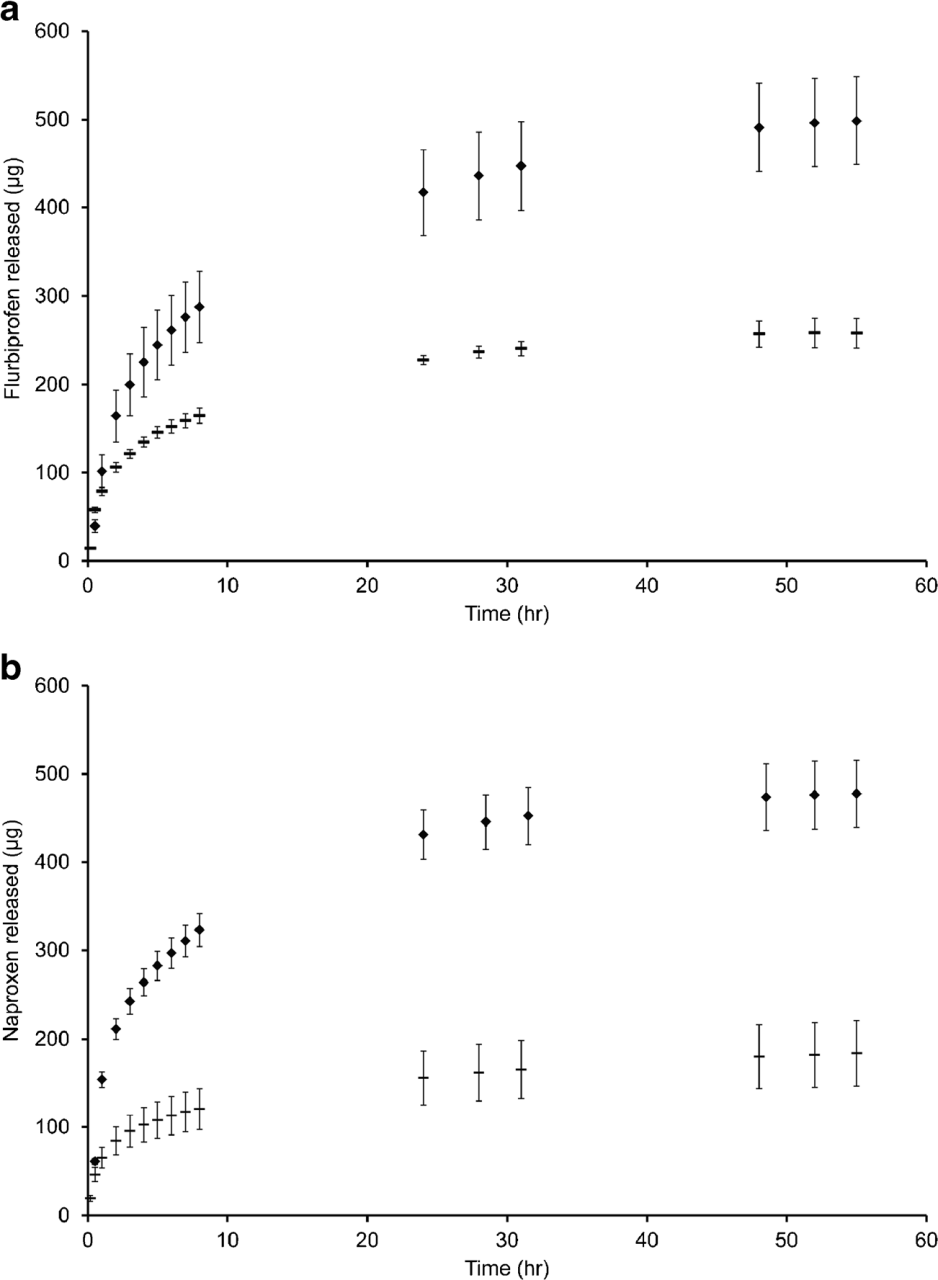
Graphs illustrating the amount of drug released following irradiation periods of 10 (−) and 30 (♦) min (mean ± st. dev, 4 replicates).

The cumulative fraction of total drug liberated from the corresponding conjugate housed in pMEA following 10 and 30 min irradiation periods is shown in Fig. 4. Following irradiation, drug is liberated from the conjugate and free to diffuse out of the pMEA matrix with the amount of drug clearly controlled by the longevity of UV exposure.

### Multi-Dosing Capacity

**Fig. 5:**
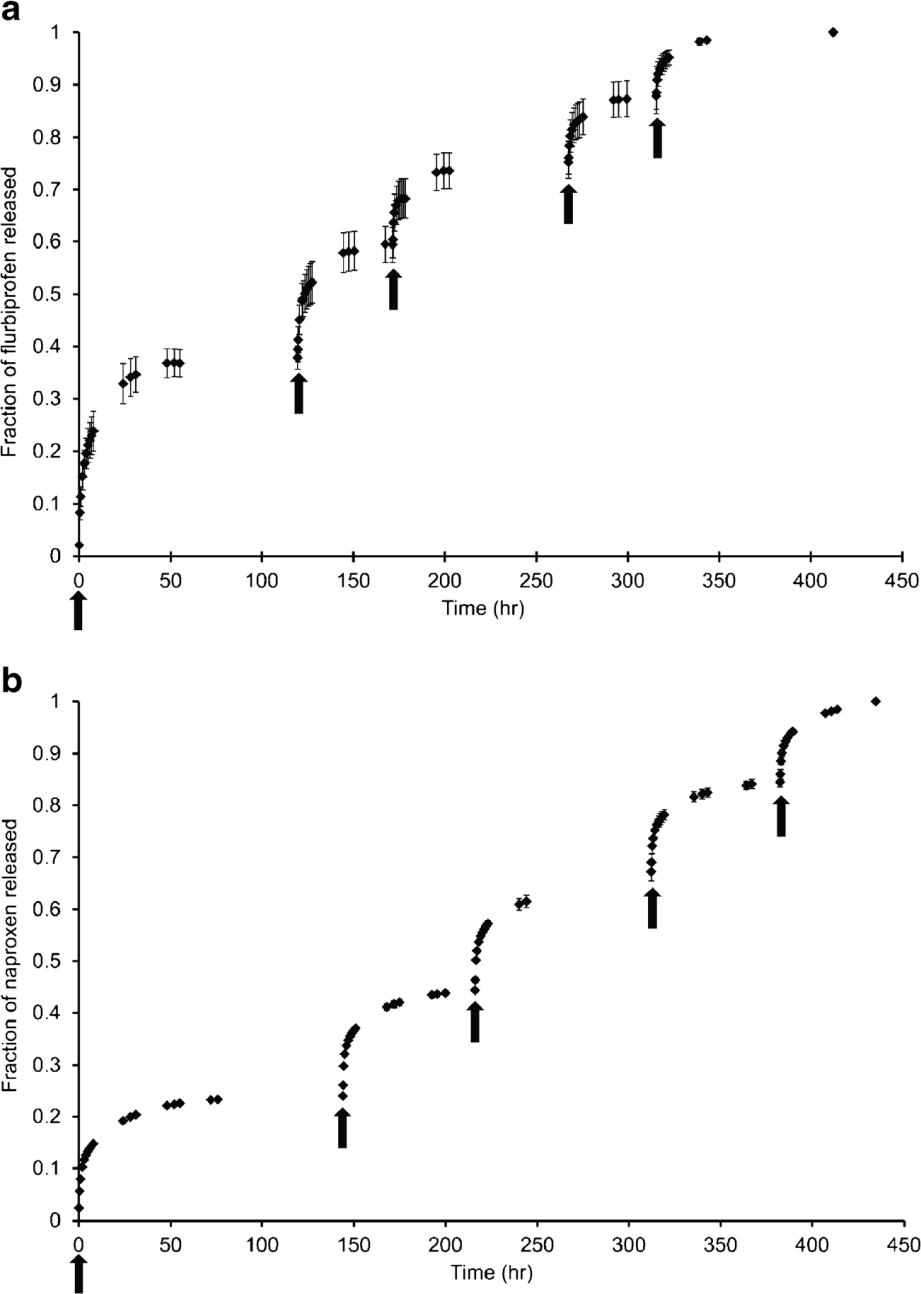
Cumulative fraction of total photo-liberated drug released from pMEA conjugate matrix following successive periods of 365 nm irradiation as indicated by arrows (mean ± st. dev, 4 replicates).

The cumulative fractional release of total flurbiprofen and naproxen liberated from the corresponding conjugate housed in pMEA during successive periods of irradiation is shown in Fig. 5. Following different periods of irradiation, the drug dose liberated is clearly controlled by the application of 365 nm irradiation as demonstrated through the stepped profile of the graph. Subsequently, the liberated dose is released from the pMEA matrix by diffusion. Photolysis rates of **2** and **3** while embedded in the polymer matrix were calculated from plots of ln[A]_t_/[A]_0_ as a function of irradiation time. The conjugate photolysis rates were determined as 5.6 x 10^−2^ and 5.5 x 10^−2^ min^−1^ respectively in pMEA.

## Discussion

This work describes the application of UV light to trigger drug release from a biomaterial coating which was prepared by the incorporation of synthesised photoactive drug conjugates into pMEA-based hydrogels. Each of the photochemical conjugates were synthesised in good yield via DCC mediated or direct acid chloride esterification reactions. Conjugates were characterised using mass spectrometry and NMR spectroscopy in which a split in some proton signals was observed due to the use of a drug racemate in each synthesis, and the corresponsing diastereoisomeric character of the photoactive compounds.

To investigate the photochemical behaviour of the synthesised conjugates upon exposure to UV light and to assess the ability and rate at which a drug dose can be generated on demand, solutions of **2** and **3** were prepared in acetonitrile and exposed to increasing periods of UV irradiation. Fig. 3 illustrates the changes in acetonitrile solutions of the conjugates as the photochemical reaction progresses. Light exposure was shown to lead to depletion of the drug conjugate (decreasing band at 246 nm) while benzofuran formation and simultaneous drug regeneration was apparent through the increasing absorption band at 300 nm. In each case, the presence of isosbestic points at 233 nm and between 262–264 nm demonstrates the clean nature of the photolysis reaction. The rate of photolysis is analogous to the rate of benzofuran formation and hence can be calculated from the rate of the appearance of the 300 nm absorption band. Both reactions were shown to follow first order reaction kinetics and rate constants were calculated as 4.6 x 10^−3^ and 7.7 x 10^−3^ s^−1^ for 2 and 3 respectively. The more rapid photodissociation of the naproxen ester in comparison to its flurbiprofen counterpart may be explained by the extended π-conjugation that exists in this molecule, allowing higher excitation through more efficient absorption of 365 nm. This work has confirmed the ability to rapidly liberate drugs from the conjugates using UV irradiation, the amount of which is controlled by the duration of light exposure.

Successful UV triggered drug regeneration in solution warranted conjugate inclusion in a polymeric scaffold in order to investigate the potential for triggered release of drug from a biomaterial matrix. Careful consideration of the choice of matrix is essential to ensure the retention of the drug-DMB conjugate and photo-generated benzofuran side product while facilitating the release of the drug. In previous studies (11,12), a copolymer of HEMA and methyl methacrylate cross linked with EGDMA met these requirements by allowing drug to diffuse into surrounding medium upon irradiation yet retain the conjugate and the photochemical byproduct while in the hydrated state. With increasing markets for biomedical devices, materials that display high biocompatibility are increasing sought for their manufacture or, alternatively, surface modification. As such, the ability of pMEA to act as a successful housing for the photoactive conjugates was examined. pMEA is an amphiphilic polymer (16) comprised of a hydrophobic polyethylene chain and a mildly hydrophilic tail which is used commercially as a coating for artificial organs (17). The hydrophilicity of this polymer is reportedly lower than that of the pHEMA material used in our previous study as determined by sessile drop contact angle measurements, 47° vs 33° in the hydrated state (18). As a result, pMEA may be more conducive for retention of the drug conjugates without warranting the inclusion of MMA. Materials were prepared from MEA using EGDMA (1% *w*/w) as a crosslinker and were loaded with conjugates (5% *w*/w) prior to polymerisation. The transparency of the resulting polymeric film in theory should permit light to effectively penetrate the material allowing the photoreaction to take place.

The conjugate-loaded films were soaked in aqueous media in darkness to allow any residual unpolymerised material to elute from the matrix and subsequent analysis of the wash solutions showed the absence of both drug and conjugate. This demonstrates the viability of pMEA for housing of the conjugates, preventing elution and hydrolytic cleavage of the conjugates while embedded in the polymer, and that no conjugate or drug is released in the absence of light. This entrapment of drug-conjugate is not only essential to prevent diffusion of the conjugate from the device coating when in use, but is also crucial so as to ensure that should a device require submergence in water to activate the hydrophilic coating prior to insertion, the full loading will remain intact and available for release on demand.

Under light conditions, i.e. exposure to 365 nm irradiation, the ability of pMEA to act as polymeric scaffold for light-triggered drug delivery was shown. Sole diffusion of flurbiprofen and naproxen into the surrounding release media was established by UV spectroscopy illustrating the success of the photochemical reaction within the matrix, subsequent drug release capabilities and also the retention of the benzofuran by-product of the reaction. Fig. 4 illustrates the cumulative fraction of flurbiprofen and naproxen released from pMEA following irradiation periods of 10 and 30 min. In each case, prolonging the longevity of exposure is shown to significantly increase the amount of drug released from the matrix.

The ability to deliver multi-doses of drug from the pMEA matrix was established by exposure of the system to sequential cycles of irradiation and storage in darkness. Fig. 5 illustrates the ability to induce the release of drug via the application of 365 nm irradiation, with each step in the profile of the cumulative fractional release graph illustrating the point at which a further cycle of 10 min 365 nm irradiation was applied. From the cumulative drug release data, the amount of conjugate consumed during each irradiation period was calculated and subsequently, the photolysis rate of the drug conjugates in pMEA determined from plots of ln[A]_t_/[A]_0_ as a function of irradiation time. In this case, at the end of the fifth irradiation cycle, it was assumed that all drug has been released and as such, [A]_0_, the concentration of ester at time 0, is derived from the total cumulative amount of drug released from all cycles. In pMEA, similar rates of photolysis were experienced by both flurbiprofen and naproxen DMB esters, which were calculated as 5.48 x 10^−2^ and 5.61 x 10^−2^ min^−1^ respectively. Although slower than experienced in solution as may have been anticipated, photolysis in the pMEA matrix has proved successful, proceeding in a clean manner with precise control over onset and dose of drug delivery provided through application and longevity of light exposure.

## Conclusion

The synthesis and photochemical behaviour of light-sensitive conjugates of flurbiprofen and naproxen has been examined. Through incorporation and successful retention of these drug conjugates in a pMEA scaffold, a biocompatible photo-responsive system has been developed whereby the release of a therapeutic can be triggered on demand by exposure to UV irradiation. In addition, dosing can be tailored through duration and quantity of light exposures. While we recognise that there are some safety concerns around using light, it is important to note that UVA (315–400 nm) is employed in both tanning beds and in phototherapy in the clinical to treat types of lymphoma that affect the skin, such as cutaneous T cell lymphoma or indeed other skin conditions such as eczema, psoriasis, vitiligo and graft versus host disease (15,19). Here, the use of minimally damaging 365 nm irradiation has proven to be successful and as such, the use of such a system can be foreseen in an extensive range of applications including direct application to tissue surfaces and to more indirect sites with the use of fibre optics. Such possibilities include the application of light to urinary catheters or endotracheal tubes via fibre optic probe allowing release of therapeutic agents at the intended site of action helping to minimise associated side effects. The approach presented here potentially addresses two significant hurdles which still exist in the controlled drug delivery field; the ability to control the precise location, and to control the precise dose, of drug delivered. Our approach of coupling light, which can be controlled precisely in terms of wavelength, intensity and position of application, directly to drug release in a practical drug delivery scaffold, is a significant step towards systems able to meet the “what, where and when” requirements of an ideal drug delivery system.

## Abbreviations

DCC: Dicyclohexylcarbodiimide
DCM: Dichloromethane
DMAP: Dimethylaminopyridine
DMB: 3,5-dimethoxybenzoin
DMF: Dimethylformamide
HEMA: 2-hydroxyethyl methacrylate
PBS: Phosphate buffered saline
PMEA: Poly(2-methoxyethyl acrylate)
UV: Ultraviolet
